# In vivo efficacy of endothelial growth medium stimulated mesenchymal stem cells derived from patients with critical limb ischemia

**DOI:** 10.1186/s12967-019-2003-3

**Published:** 2019-08-09

**Authors:** Rida Al-Rifai, Philippe Nguyen, Nicole Bouland, Christine Terryn, Lukshe Kanagaratnam, Gaël Poitevin, Caroline François, Catherine Boisson-Vidal, Marie-Antoinette Sevestre, Claire Tournois

**Affiliations:** 1 EA-3801, SFR CAP-santé, Université de Reims Champagne-Ardenne, 51092 Reims Cedex, France; 2 Laboratoire d’Hématologie, CHU Robert Debré, Reims, France; 3 Laboratoire d’Anatomie Pathologique, Université de Reims Champagne-Ardenne, Reims, France; 4 Plateforme PICT, Université de Reims Champagne Ardenne, Reims, France; 5 Unité d’aide méthodologique CHU Robert Debré, Reims, France; 6 Inserm UMR S1140, Faculté de Pharmacie de Paris, Paris, France; 7 Université Paris Descartes, Sorbonne Paris Cité, Paris, France; 8 Service de Médecine Vasculaire, CHU, Amiens, France

**Keywords:** Angiogenesis, Cell therapy, Critical limb ischemia, Mesenchymal stem cells

## Abstract

**Background:**

Cell therapy has been proposed for patients with critical limb ischemia (CLI). Autologous bone marrow derived cells (BMCs) have been mostly used, mesenchymal stem cells (MSCs) being an alternative. The aim of this study was to characterize two types of MSCs and evaluate their efficacy.

**Methods:**

MSCs were obtained from CLI-patients BMCs. Stimulated- (S-) MSCs were cultured in endothelial growth medium. Cells were characterized by the expression of cell surface markers, the relative expression of 6 genes, the secretion of 10 cytokines and the ability to form vessel-like structures. The cell proangiogenic properties was analysed in vivo, in a hindlimb ischemia model. Perfusion of lower limbs and functional tests were assessed for 28 days after cell infusion. Muscle histological analysis (neoangiogenesis, arteriogenesis and muscle repair) was performed.

**Results:**

S-MSCs can be obtained from CLI-patients BMCs. They do not express endothelial specific markers but can be distinguished from MSCs by their secretome. S-MSCs have the ability to form tube-like structures and, in vivo, to induce blood flow recovery. No amputation was observed in S-MSCs treated mice. Functional tests showed improvement in treated groups with a superiority of MSCs and S-MSCs. In muscles, CD31+ and αSMA+ labelling were the highest in S-MSCs treated mice. S-MSCs induced the highest muscle repair.

**Conclusions:**

S-MSCs exert angiogenic potential probably mediated by a paracrine mechanism. Their administration is associated with flow recovery, limb salvage and muscle repair. The secretome from S-MSCs or secretome-derived products may have a strong potential in vessel regeneration and muscle repair.

*Trial registration* NCT00533104

**Electronic supplementary material:**

The online version of this article (10.1186/s12967-019-2003-3) contains supplementary material, which is available to authorized users.

## Background

Critical limb ischemia (CLI) is the most severe form of atherosclerotic peripheral arterial disease (PAD). Due to the progression of vascular risk factors, PAD incidence is predicted to double by 2050 [1]. The management of patients is limited to surgical revascularization. However, around up to 30% of patients are not eligible for such procedures due to poor distal vascular bed. Therefore, these “no-option” patients (NO-CLI) experience a high risk of major amputation [2] and are exposed to a high level of cardiovascular death [3].

In such context, cell therapy (CT) has been proposed for NO-CLI patients to promote angiogenesis and improve tissue perfusion [4]. To date over 120 phase I/II or III clinical trials have investigated a variety of cell therapies [5]. Recent meta-analysis [6] are in favor of a clinical improvement of treated patients but results remain divided [7, 8]. In most cases, non-selected autologous bone marrow derived cells (BMCs) were used. They were obtained from elderly ill patients and exhibit low proangiogenic potential [9]. In most studies BMCs were not well characterized.

We recently published a multicenter clinical trial evaluating the effect of autologous BMCs versus placebo in CLI-patients [Bone Marrow Autograft in Limb Ischemia (BALI)]. This study was in favor of efficacy but the rate of amputation remained elevated in treated patients [10]. Inflammation was associated with poor outcome [11]. In order to improve our understanding, BMCs were extensively characterized and we observed a great variability in BMCs composition between patients [11].

BMCs contained mature and immature cells [hematopoietic stem cells (HSCs), endothelial progenitor cells]. Interestingly, they also contained a rare subset of mesenchymal stem cells (MSCs). These non-hematopoietic mononuclear cells reside in the bone marrow (BM) stroma [12]. MSCs can be obtained in adults from many tissues such as adipose tissue (ADSCs), peripheral blood, synovial membrane and dental pulp. Fetal/neonatal tissues [e.g., umbilical cord blood, umbilical cord Wharton’s jelly (WJ-MSCs), amniotic fluid, placenta] are also a potential source of MSCs [13]. Still, human BM remains the main source of MSCs. They show an extensive capacity of differentiation into osteoblasts, chondrocytes, adipocytes, astrocytes and skeletal muscle cells [14]. MSCs can migrate, proliferate in areas of ischemia and can promote regeneration of damaged tissues and reducing inflammation [15]. They may be good candidates for CT as they combine proangiogenic, anti-inflammatory and immunomodulatory properties [16].

CT protocols in CLI-generally require hundreds of millions of MSCs per treatment [17–19]. Therefore, in vitro cell expansion is needed. In autologous situation, the patient’s age and clinical characteristics influence the culture efficiency [20]. Major efforts have been made to improve culture conditions and favor endothelial induction by adding supplements containing pro-angiogenic factors [vascular endothelial growth factor (VEGF), epidermal growth factor (EGF), fibroblast growth factor-2 (FGF2) and insulin-like growth factor-1 (IGF-1)] [21–27]. The so called “endothelial cell-specific growth medium” (EGM-2) improves the proliferation rate and may induce the acquisition of endothelial markers. These stimulated-MSCs (S-MSCs) can form functional blood vessels in collagen-plug implanted in mice [21] but have never been tested in a hind limb ischemia model (HLIM).

The purpose of this study was: (1) to evaluate the presence of MSCs in CLI-patients BMCs, (2) to analyze the phenotype of S-MSCs, and (3) to compare the effect of MSCs and S-MSCs in a murine HLIM in comparison with BMCs.

## Methods

### Source and characterization of BMCs

BMCs were obtained from CLI patients included in the BALI multicenter trial (trial number NCT00533104). The study protocol was approved by the French National Agency for Medicines and Health Products Safety and by an Institutional Review Board. As previously described [10], 500 mL of BM were collected under general anesthesia through multiple punctures of the posterior iliac crest. BMCs were isolated using a blood cell separator (Cobe Spectra, version 4, BM Processing Program, Gambro BCT, Lakewood, CO, USA) and concentrated to obtain a final volume of 40 mL (30 mL for autologous injection and 10 mL for control analyses and cryopreservation). BMCs were cryopreserved in liquid nitrogen in dimethyl sulfoxide as a cryoprotector.

The present study is as an ancillary study of BALI. BMCs were exclusively provided by the centers of Reims and Amiens (out of 9 centers) as they used similar methods of cell preparation, preservation and storage. Seven CLI-BMCs were selected regardless of patients’ characteristics or clinical outcome.

BMCs were extensively characterized. For this, platelets (PLTs) and nucleated cells counts were performed with an Advia 2120 automated counter (Siemens, Health care SAS, Saint-Denis, France). Cell morphology was observed after cytocentrifugation and May Grünwald Giemsa staining (Fig. 2a). CD34+ stem cell analysis was performed by flow cytometry (FC) according to the ISHAGE (International Society of Hematotherapy and Graft Engineering) reference method using Stem-Kit reagents (Beckman Coulter, Villepinte, France). Samples were analyzed on a Navios flow cytometer (Beckman Coulter). The MesenCult Proliferation Kit (Stemcell Technologies, Grenoble, France) was used for the fibroblast colony-forming units (CFU-F) assay.

### Isolation of BM-MSCs and cell culture

**Fig. 1:**
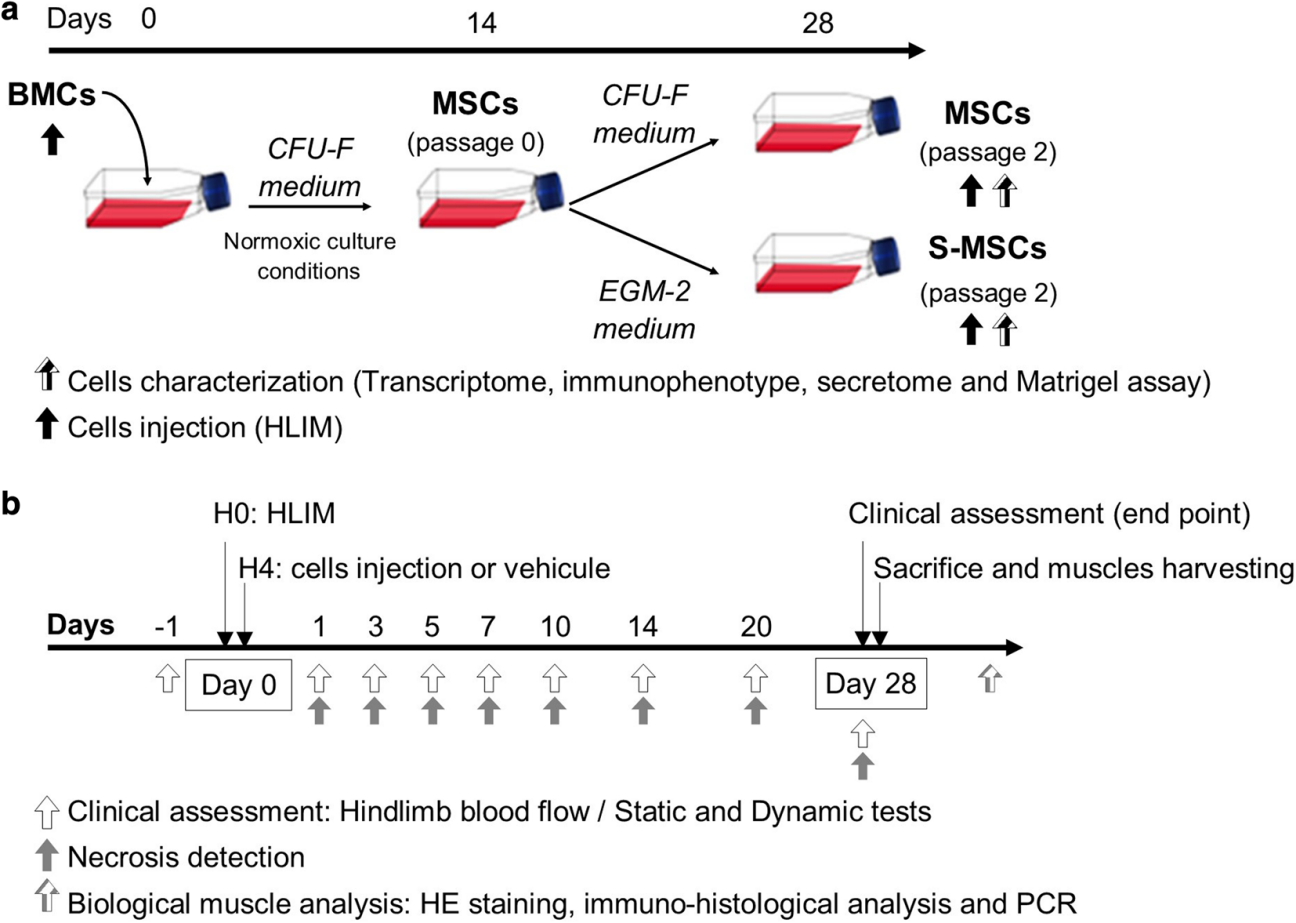
Study design. **a** BMCs were obtained from 7 CLI patients. MSCs were selected and expanded in a CFU-F medium for 28 days (n = 7). Stimulated MSCs were cultured in EGM-2 medium for 14 days (n = 7). **b** Hindlimb ischemia was induced by femoral artery ligation in *Nude* mice. Cells (BMCs, MSCs, S-MSCs) or vehicle were injected in the *gastrocnemius*. Hindlimb perfusion, Necrose detection and functional tests were performed during 28 days. At day 28 muscles were harvested for biological analysis

MSCs were obtained from 7 CLI cryopreserved BMCs. After thawing, cells were counted, and viability was evaluated. MSCs were selected by their capacity to adhere to uncoated plastic plates, cultured and expanded in a CFU-F medium (MesenCult™ Proliferation Kit, Stemcell Technologies) supplemented with 1% penicillin–streptomycin–amphotericin (Antibiotic–Antimycotic 100X, Gibco by Life Technologies, Illkirch, France). Cells were cultured in a humidified incubator at 37 °C under a 5% CO_2_ atmosphere. The culture was maintained through 28 days (Fig. 1a).

S-MSCs were obtained from 7 CLI-MSCs after trypsinization at day 14, and subsequent culture during another period of 14 days in a Endothelial growth medium (EGM)-2 comprised endothelial basal medium (EBM)-2 supplemented with SingleQuots Bulle-kit (Lonza, Basel, Switzerland) (0.5 ng/mL human VEGF, 5 ng/mL human EGF, 10 ng/mL human FGF2, 20 ng/mL long R3-insulin-like growth factor-1, 22.5 µg/mL heparin, 1 µg/mL ascorbic acid, 0.2 µg/mL hydrocortisone) supplemented with 1% penicillin–streptomycin–amphotericin (Antibiotic–Antimycotic 100X, Gibco). Cells were cultured in a humidified incubator at 37 °C under a 5% CO_2_ atmosphere (Fig. 1a).

Culture media were changed every 3 or 4 days. MSCs and S-MSCs were observed in phase contrast microscopy (Olympus, Plateforme en Imagerie Cellulaire et Tissulaire PICT). Before injection, cells were washed twice in Phosphate Buffer Saline (PBS) (ET330, Euromedex, Souffelweyersheim, France), then counted. Viability was determined by Trypan blue and was above 90 ± 6%. Cells were resuspended in PBS.

### Characterization of MSCs and S-MSCs

#### Transcriptomic characterization of MSCs, S-MSCs and cb-ECFCs by simultaneous real-time qPCR reactions

Gene expression analysis was performed at day 28, on both MSCs (n = 7) and S-MSCs (n = 7). Cord blood endothelial colony-forming cells (cb-ECFCs, n = 5) were used as control. Isolation and characterization of cb-ECFC were performed as previously described [28]. Total RNA was extracted from 5 × 10^6^ cells, using the RNeasy Mini kit (Qiagen, Courtaboeuf, France). RNA was treated to eliminate any DNA contamination (Qiagen) and the quality of mRNA was assessed using the Experion automated electrophoresis system (Bio-Rad, Marnes La Coquette, France). Complementary DNA was synthesized using the high-capacity cDNA reverse transcriptase (RT) kit with RNase inhibitor (RT^2^ HT First Strand Kit, Qiagen). PCR primers targeting von Willebrand factor (vWF)(NM_000552.3), platelet endothelial cell adhesion molecule 1 (PECAM1)(NM_000442.4), vascular endothelial growth factor receptor 2 (VEGF R2 or KDR)(NM_002253.2), Ve-Cadherin (CDH5)(NM_001795.3), CXC motif receptor 4 (CXCR4)(NM_003467.2), vascular cell adhesion molecule 1 (VCAM1)(NM_001078.3), beta-2 microglobulin (B2M)(NM_004048), hypoxantine phosphoribosyltransferase-1 (HPRT1) (NM_000194) and glyceraldehyde-3-phosphate-dehydrogenase (GAPDH)(NM_002046) were obtained from Qiagen (RNA QC PCR Array Qiagen). B2 M, HPRT1 and GAPDH were used as housekeeping genes. Conventional PCR was performed under standard conditions (RT^2^ SYBR Green ROX qPCR, Qiagen) and analyzed on the ABI 7500 FAST-Real Time PCR System (Thermo Fisher Scientific, Courtaboeuf, France). Assays were systematically run in duplicate for each type of cells and for internal controls (human genomic DNA contamination, reverse transcription control and positive PCR control). The mRNA levels of vWF, PECAM1, VEGF R2, Ve-Cadherin, CXCR4 and VCAM1 were normalized to housekeeping genes using the 2^−ΔCt^ method (= 2^−[Ct(target) − Ct(housekeeping gene)]^).

#### Flow cytometry analysis

A four-color FC analysis was performed on FC500 analyzer (Beckman Coulter) to characterize MSCs and S-MSCs at day 28: FITC-conjugated CD105 (endoglin), CD31 (PECAM1) antibodies and PE-conjugated CD90 (Thy-1), CD140a (Platelet-derived growth factor receptor alpha, PDGF RA), CD140b (Platelet-derived growth factor receptor beta, PDGF RB), CD144 (Ve-Cadherin, CDH5), VEGF R1 (Vascular endothelial growth factor receptor 1, FLT1) antibodies and PE-Cy5-conjugated HLA-DR (human leukocyte antigen-D related), CD34, CD45, CD11b (integrin alpha M), CD146 (Melanoma adhesion molecule, MCAM), CD184 (CXCR4), CD106 (VCAM1) antibodies and PE-Cy7 conjugated CD73 (5′ nucleotidase) and VEGF R2 (KDR, CD309) antibodies. Mouse anti-Human CD105, CD90, CD73, HLA-DR, CD140a, CD31, CD184, CD106 were obtained from BD Biosciences (Le Pont de Claix, France), CD34, CD45, CD11b, CD146, VEGFR2 from Beckman Coulter, CD140a, VEGFR1 from R&D Systems Inc (Minneapolis, USA) and CD144 from Santa-Cruz Biotechnology inc SCBT (Dallas, USA). The isotype-matched mouse IgG1-FITC, IgG1-PE, IgG1-PCy5 and IgG1-PCy7 were used as negative controls. Acquisition and processing data from 7,000 events were analysed using Kaluza software.

#### Cell secretome

Cell secretome was characterized by the quantification of growth factors productions and by their capacity to form tube-like structures.

#### Preparation of MSCs- and S-MSCs-derived conditioned medium

Culture media (CFU-F and EGM-2) had been changed at day 24. Aliquots of the CFU-F and EGM-2 media free of cells were placed in a humidified incubator at 37 °C under a 5% CO_2_ atmosphere during 4 days. MSCs (n = 7) and S-MSCs (n = 7) culture supernates and cell-free media (CFU-F and EGM-2) were recovered at day 28, centrifuged at 300*g* for 5 min, filtered through a 0.22 µm and were then aliquoted and stored frozen at − 40 °C until use.

#### Growth factors assays

A set of ten growth factors [VEGF-A, EGF, FGF2, IGF-1, Angiopoietin-1 (Angio-1), Interleukin-6 (IL-6), HGF, Platelet-derived growth factor alpha polypeptide (PDGF-AA), Leukemia-inhibitory Factor (LIF), Chemokine CXC motif Ligand 12 (CXCL12 or Stromal-cell-derived factor-1, SDF-1)] was measured in the MSCs and S-MSCs culture supernates at day 28. Quantitative determination of IGF-1 concentrations was performed using the Quantikine ELISA kit (R&D Systems Inc). The 9-plex LEGENDplex panel is a bead-based multiplex assay panel, using fluorescence–encoded beads suitable for use on LSRFortessa (BD Biosciences). This panel allows the simultaneous quantification of 9 human cytokines (VEGF-A, EGF, FGF2, Angio-1, IL-6, HGF, PDGF-AA, LIF, CXCL12) (BioLegend Ozyme, Saint Quentin en Yvelines, France) (Plateau technique de Cytometrie en flux URCACyt). The Bradford Protein assay (Quick Start™ Bradford 1× Dye Reagent, Bio-Rad) was used to measure protein quantification in MSCs and S-MSCs culture supernates and in 2 media (CFU-F and EGM-2).

#### Tube formation assay

In order to assess the angiogenic effect of culture supernates, an in vitro assay was performed evaluating tubule formation from HMEC-1 endothelial cell line (Dermal microvascular endothelium, ATCC CRL-3243) [29]. HMEC-1 cells (8000 cells/well) were suspended in Endothelial Cell Basal medium MV (n = 3) (10 ng/mL EGF, 1 µg/mL hydrocortisone, 10 mM Glutamine, and 10% FBS) (PromoCell, Heidelberg, Germany), or in cell-free media [CFU-F (n = 3), or EGM-2 (n = 3)] or in MSCs culture supernates (obtained from 5 CLI-MSCs, n = 2 each group), or in S-MSCs culture supernates (obtained from 5 CLI-S-MSCs, n = 2 each group), laid upon Matrigel (BD Biosciences, Le Pont de Claix, France) cast in IBIDI micro wells (81,501, µ-Slide Angiogenesis, Biovalley), and allowed to form tubules for 24 h under normoxic conditions. Slides were observed during 24 h with a videomicroscope (Axiovert, 200 M, Zeiss, Germany) piloted by Software Metamorph (Roper Scientific). Pictures were catched each 15 min (coolsnap HQ, Roper Scientific, France). The capillary-like tubes were appreciated at 3:30 h by the quantification of the loops number and total tube length using the ImageJ software and Neuron-J plug in tool.

#### Cell functional assay: in vitro tube formation assay

Capillary-like tube formation was evaluated in a Matrigel (BD Biosciences) matrix. A volume of 10 µL of gel was deposited on the uncoated slide (81,501, µ-Slide Angiogenesis, Biovalley). After 30 min at 37 °C, a gel was formed and 10^4^ cells (MSCs, S-MSCs, MRC and cb-ECFC) were deposited. The extend of the network of the capillary-like tubes was appreciated at the time of maximal network (as indicated on Additional file 1: Figure S1) by the quantification of the loops number using the ImageJ software and Neuron-J plug in tool. MRC5 fibroblasts cells line (ATCC CCL-171; RD-Biotech, Besançon, France) was used as negative control. cb-ECFC were used as positive control.

### Animal study

#### Study design

The aim of the study was to evaluate the efficacy of BMCs, MSCs and S-MSCs from CLI patients in comparison with vehicle in a murine HLIM (Fig. 1b). BALB/c *Nude* mice were included in the study. Hindlimb perfusion and functional tests were assessed at baseline (day-1). At day 0 hindlimb ischemia was induced after left common femoral artery ligation. Blood flow of the lower limbs was performed postoperatively. Viable cells [BMCs or MSCs or S-MSCs) or vehicle (PBS)] were injected in the left g*astrocnemius* muscle 4 h after ischemia induction. Blood flow measurement was considered as primary endpoint whereas necrosis detection and functional tests (static and dynamic) were considered as secondary endpoints. They were evaluated at days 1, 3, 5, 7, 10, 15, 20 and 28 postoperatively. At day 28 *gastrocnemius* and *semimembranosus* muscles were harvested for immunohistological analyses (Fig. 1b).

#### Murine hindlimb ischemia model and cell transplantation

One hundred and thirty-five male BALB/c *Nude* mice (Charles River Laboratories, l’Arbresle, France), 11 weeks of age were used for all experiments. They had access to water and food ad libitum. Hindlimb ischemia was induced after the surgical left common femoral artery ligation [30]. Animals were placed on a 38 °C heating pad and anesthetized by continuous inhalation of isoflurane (5% in induction and 2% during the intervention). Exposure was obtained by performing an incision in the skin overlying the high portion of the left hindlimb next to the inguinal ligament. The common left femoral artery was ligated with a non-absorbable 6.0 silk thread. Then the skin was closed with absorbable sutures. Buprenorphine was administered as analgesic (0.05 mg/kg twice a day for 3 days). Under anesthesia, mice were transplanted in their left *gastrocnemius* 4 h after surgery with 30 µL of PBS (n = 20 mice) or with 30 µL of 5 × 10^5^ BMCs (n = 40 mice and approximately 6 per condition) or of 5 × 10^5^ MSCs (n = 35 mice and approximately 6 per condition) or of 5 × 10^5^ S-MSCs (n = 40 mice and approximately 6 per condition). The quantity of injected cells (5 × 10^5^) can be considered as low in comparison with similar previously published studies [31–33]. A 30-gauge needle was used for the unique intramuscular injection.

#### Blood flow analysis

Perfusion of lower limbs was assessed with PeriCam^®^ perfusion imaging (PeriCam^®^ PSI, Perimed, Craponne, France) at baseline and for 28 days after ligation. The Pericam perfusion imaging was able to quantify the blood flow in the microcirculation (arterioles, capillaries and veins) and the laser speckle contrast analysis (LASCA) facilitated high resolution and real-time visualization. Measures were taken under anesthesia with isoflurane and on a 38 °C heating pad. Low or no blood perfusion was displayed as dark blue, whereas the highest perfusion was displayed as red. After blood flow was scanned during 30 s, stored records were analyzed with PimSoft^®^ software and the average flows of ischemic and non-ischemic limbs were quantified. Ratios of the ischemic (left)/normal (right) limb blood flow were used to express the results. The mean and the standard deviation were used per group.

#### Clinical recovery

*Necrosis detection* Limb ischemia was assessed with the daily observation of mouse limbs searching for nail, toe, foot and leg necrosis or amputation.

Two functional tests (static and dynamic) were used to quantify post-ligature clinical improvement:

- *Static test*: After surgical ligation, ischemic mouse paw retracted. Interdigital spacing (IDS) between the first and the 5th toe was measured using Image J software. For this, the mouse was placed in a transparent container to visualize from the bottom normal and ischemic paws. Measures were taken at day-1 (baseline), 1, 3, 5, 7, 10, 14, 20 and 28 post surgery. Results were expressed as a ratio of IDS ischemic/control paw. Clinical improvement was testified by the gradual recovery of IDS.
- *Dynamic test*: A semi quantitative assessment of impaired use of ischemic limb was performed. Mice progressed in a transparent corridor to evaluate their walking. The functional scoring was adapted from Tarlov‘s [31]: score 0: the mouse does not use the paw-score 1: the mouse can rely on the left paw-score 2: the mouse can use its left paw but claudicates-score 3: normal walking. Measures were taken at day-1 (baseline), 1, 3, 5, 7, 10, 14, 20 and 28 post surgery.

The mean and the standard deviation were used per group.

#### Tissue preparation and immunohistological analysis

The *gastrocnemius* and the *semimembranosus* muscles [30] right and left of each mouse were harvested at day 28. The *semimembranosus* muscle is localized close to the ligation upstream from the cell infusion site. For each condition, the muscles were frozen for immunofluorescence assay or fixed in formalin 10% for hematoxylin eosin (HE) staining.

For immunofluorescence, tissues were embedded in Optimal Cutting temperature (OCT) compound (F/KMA-0100-00A, MM France, Francheville, France) and frozen in liquid nitrogen. Frozen sections of 6-µm thickness were mounted on superfrost slides. Sequentially, slides were fixed in cold acetone for 20 min, blocked with normal donkey serum (S30, Merck Millipore, St-Quentin-en-Yvelines, France) for 1 h at room temperature and incubated with primary antibody at 4 °C overnight. After washing three times with PBS, slides were incubated with secondary antibody during 1 h at room temperature. The vessels were visualized by immunofluorescent staining with a first anti-CD31 antibody (ab28364, abcam, Cambridge, United Kingdom) and a second anti alpha-smooth muscle actin antibody (αSMA) (ab150129, abcam). Secondary fluorochrome-coupled antibodies were used (Alexa Fluor^®^ 568, ab175470, abcam) and (Alexa Fluor^®^ 488, ab21027, abcam). Slides were observed on an inverted fluorescence microscope (20× magnification Axio Observer Z1, Zeiss Microscopy) piloted by Metamorph Software (Roper Scientific). Capillaries and arterioles were counted in 5 representative fields from 3 tissue sections for each muscle using ImageJ software. The amount of ischemia-induced angiogenesis and arteriogenesis were compared with the non-ischemic muscle. Macrophage infiltration were visualized by the same protocol using an anti-CD68 antibody (ab125212, abcam).

The fixed *gastrocnemius* and *semimembranosus* muscles were embedded in paraffin and stained with HE. We determine the percentage of muscle fibers with a central nucleus. Indeed, these muscle fibers specifically indicate muscle regeneration [34, 35]. For this, five fields were counted in the central region of the largest cross area *of gastrocnemius* muscle sections. The results were expressed as a ratio left muscle/right muscle. All results were expressed as mean and standard deviation per group.

#### Real time quantitative polymerase chain reaction (qPCR)

Real time qPCR was performed on right and left harvested *gastrocnemius* muscles of 4 mice (1 mouse for each group: vehicle, BMCs, MSCs and S-MSCs) at day 28 in order to detect human DNA. The DNA was extracted from the muscles with a DNeasy Blood and Tissue kit (69504, Qiagen) according to the manufacturer’s instructions. Human DNA (n = 2) was used as positive control. *Gastrocnemius* muscles of vehicle group and non-ischemic controlateral muscles were used as negative control. The qPCR was performed for human Factor V gene (TaqMan SNP genotyping assay, C_11975250_10, Thermo Fisher Scientific) and with a TaqMan universal PCR master mix (4324018, Thermo Fisher Scientific). We checked that human Factor V primers did not hybridize with mouse DNA using BLAST^®^. Each DNA was analyzed thrice. The limit of detection of PCR was defined by a threshold cycle (Ct) > 35.

### Statistical analyses

Quantitative variables were expressed as mean and standard deviation per group or as median (Med) and range [min–max] or Box and Whiskers plots (Medcalc version 7.3 software, https://www.medcalc.org/). Mann–Whitney Tests were used to compare differences between groups at day 28. Wilcoxon Tests were used to compare differences between days in the same group (Statview^®^). A value of p < 0.05 was considered statistically significant. For blood flow analysis, a mixed effect model was performed to assess the relationship between the ischemic/normal limb ratio and treatment group, the time of the measure, adjusted on the value of the ratio at day-1 (baseline value). Bonferroni method was used for multiple comparisons adjustments. The overall survival limb rates were estimated by the Kaplan–Meier method. Difference between survival curves for each experimental group was performed using the log rank test (GraphPad^®^).

## Results

### Patient and BMCs characteristics

BMCs were obtained from 7 different patients presenting with CLI and candidates for CT. Patients were representative of the NO-CLI population in terms of age, associated cardiovascular risk factors, and current medications (Table 1). The severity of the disease was appreciated from percutaneous tissue oxygen pressure (TcPO_2_) and the presence of ulcers.

**Table 1:** Patient characteristics

	1	2	3	4	5	6	7
Patients characteristics							
Age (years)	74	57	52	57	74	62	42
Gender (M: male; F: female)	M	M	M	M	F	M	M
BMI (kg/m^2^)	27	25	26	32	20	22	21
Smoker	No	Past	Past	Past	No	Past	Current
Arterial hypertension	Yes	Yes	No	No	No	Yes	No
Hyperlipidemia	Yes	No	Yes	No	Yes	No	No
Diabetes mellitus	Yes	No	Yes	No	No	No	No
Disease characteristics							
TcPO_2_ (mmHg)	26	30	64	35	45	1	7
Presence of ulcers	No	No	Yes	No	No	No	No
Cardioprotective drugs use^a^	AT, ACEI	AT, ACEI, S	AT, ACEI, S	AT, ACEI, S	AT	AT, S	AT, S
BMC characterization							
PLTs (G/L)	746	740	478	590	384	1296	1118
Total nucleated cells (G/L):	47	60	38	48	50	76	71
Total MNCs							
Lymphocytes (%)	46	47	39	41	48	46	54
Monocytes (%)	18	12	28	18	19	14	13
Erythroblasts and other cells (%)^b^	10.5	14.3	10.4	19.8	12.7	13.2	14.5
Mature granulocytes (%)	23	23	18	16	17	23	16
HSCs (CD34+ cells) (%)	2.5	3.7	4.6	5.2	3.3	3.8	2.5
MSCs^c^	42	38	34	58	28	12	60
Placebo or BMCs group	BMCs	BMCs	Placebo	Placebo	BMCs	BMCs	Placebo
Clinical outcome at 6 months	∅	∅	∅	∅	∅	Revascularization at day 126	Major amputation at day 114
Clinical outcome at 12 months	Revascularization at day 332	∅	∅	∅	∅		

Quantitative variables are expressed as median or absolute value. Biologic markers measured in 7 NO-CLI patients on day 0 (baseline) before CT

∅: Patient alive without any major amputation

^a^Cardioprotective drugs use: antiplatelet therapy (AT), statins (S), angiotensin-converting enzyme inhibitor (ACEI)

^b^Other cells: blasts, immature granulocytes and plasma cells

^c^CFU-F number/1 × 10^6^ nucleated cells

**Fig. 2:**
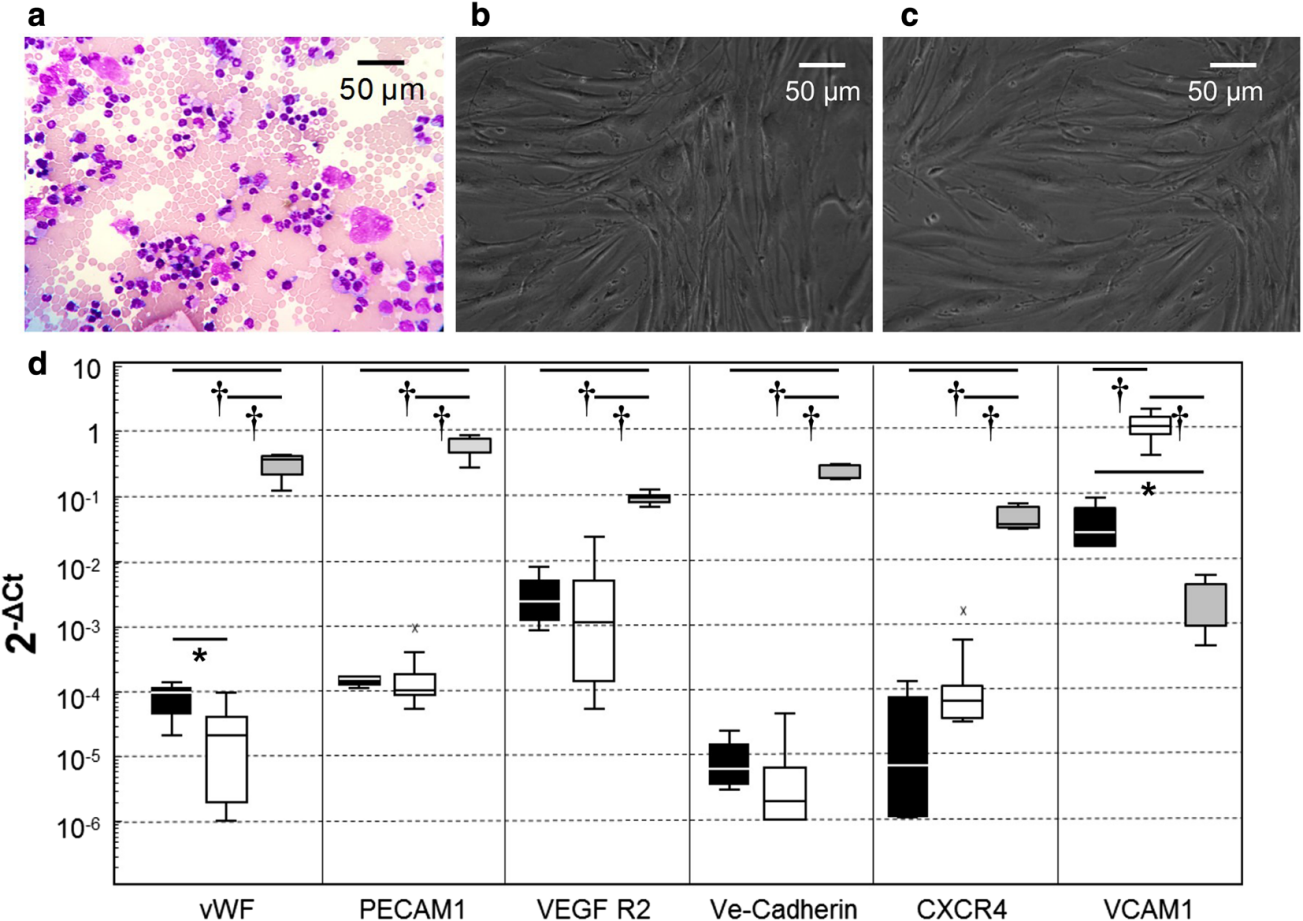
Cells observation and real-time RT-PCR analysis. **a** BMCs morphology in May Grünwald Giemsa staining. **b** MSCs morphology in phase contrast microscopy. **c** S-MSCs morphology in phase contrast microscopy. **d** mRNA expression of six genes of interest were quantified using simultaneous real-time RT-qPCR experiments in MSCs (black box, n = 7), in S-MSCs (empty box, n = 7) and cb-ECFC (grey box, n = 5) used as control. x: aberrant distribution values as indicated by Box and Whiskers plots Medcalc version 7.3 software (*p < 0.05 and ^†^p < 0.01)

**Fig. 3:**
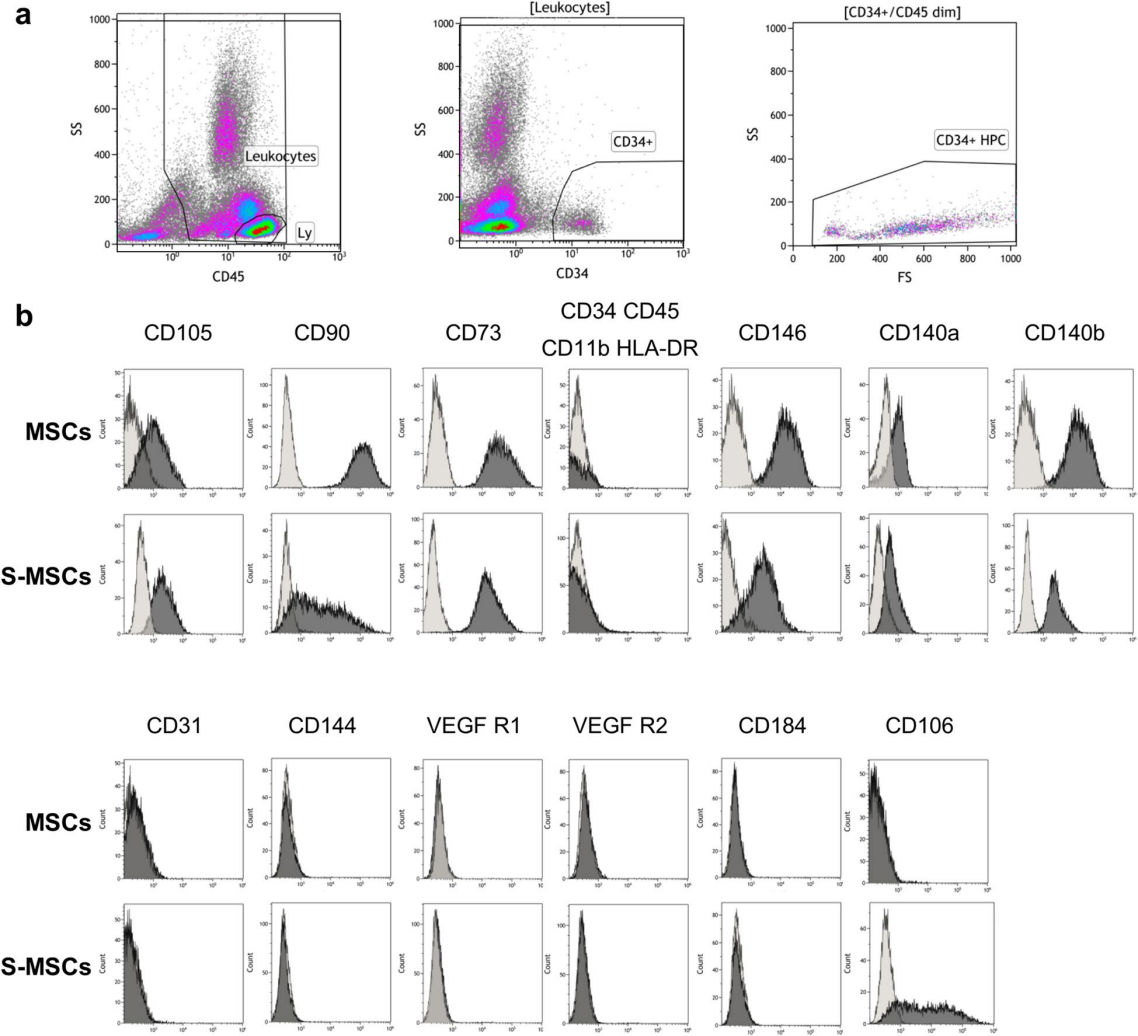
Cells characterization by FC. **a** BMCs characterization. HSCs (CD34+ cells) analysis was performed by FC according to the ISHAGE reference method. **b** Immunophenotype characterization of isolated MSCs and S-MSCs. MSCs and S-MSCs (grey) were stained with the following human monoclonal antibodies: CD105 (endoglin), CD90 (Thy-1), CD73 (5′ nucleotidase), CD34, CD45, CD11b (integrin alpha M), HLA-DR (human leucocyte antigen-D related), CD146 (MCAM), CD140a (PDGF RA), CD140b (PDGF RB), CD31 (PECAM1), CD144 (Ve-Cadherin), VEGF R1, VEGF-R2, CD184 (CXCR4) and CD106 (VCAM1). The isotype-matched mouse IgG1-FITC, IgG1-PE, IgG1-PCy5 and IgG1- PCy7 were used as negative controls (empty)

BMCs were characterized by cell counting and differential presented in Figs. 2a, 3a and Table 1. HSCs were quantified in BMCs by FC: 3.66% ± 1% (Fig. 3a). MSCs were quantified in BMCs by a clonogenic assay (CFU-F): 39 ± 16 MSCs of 1 million nucleated cells (Table 1).

Clinical outcome after 6 and 12 months is presented in Table 1.

### MSCs and S-MSCs in vitro characterization

#### Cells morphology

Human BM derived MSCs show plastic adherent properties and have fibroblast-like morphology seen under phase contrast microscope (Fig. 2b). S-MSCs were maintained in EGM-2 medium from subculture 0 to 2 during 14 days. S-MSCs presented similar fibroblast-like morphology (Fig. 2c). The S-MSCs’ doubling time was significantly shorter in comparison with MSCs (respectively 4.6 ± 1.6 versus 7.3 ± 1.8 days, p < 0.01).

#### Transcriptomic profile

In order to differentiate S-MSCs from MSCs, we determined the transcriptomic profile of both cells. For this, the expression of endothelial-lineage genes (vWF, PECAM1, VEGF R2, Ve-Cadherin, CXCR4 and VCAM1) was examined in MSCs and S-MSCs at day 28 (Fig. 2d). cb-ECFCs was used as a endothelial cells (ECs)-control. A set of four genes (PECAM1, VEGF R2, Ve-Cadherin and CXCR4) was expressed in a comparable way in both MSCs and S-MSCs. S-MSCs expressed a significant, 44-fold higher level of VCAM1 in comparison with MSCs. In contrast, S-MSCs expressed a significantly lower level of vWF (4.1-fold lower) in comparison with MSCs. In cb-ECFCs, the level of expression of vWF, PECAM1, VEGF R2, Ve-Cadherin and CXCR4 was significantly higher in comparison with MSCs and S-MSCs.

#### Phenotype of isolated MSCs and S-MSCs

The cultured MSCs express on their surface CD105+, CD90+ and CD73+, while lacking the expression of hematopoietic cell markers (CD45−, CD34−, CD11b−) and HLA-DR surface marker (Fig. 3b). The MSCs were strongly positive for CD146 (MCAM), CD140b (PDGF RB), weakly positive for CD140a (PDGF RA) and negative for endothelial-specific markers (CD31, CD144, VEGF R1, VEGF R2), CD184 (CXCR4) and CD106 (VCAM1).

S-MSCs grown in EGM-2 during 2 weeks showed the classic pattern of MSCs (CD105+, CD90+, CD73+, CD45−, CD34−, CD11b− and HLA-DR−) but did not express endothelial-specific markers (CD31, CD144, VEGF R1, VEGF R2). S-MSCs retained the expression of CD90 and acquired the CD106 marker (VCAM1) (Fig. 3b). For both markers, a double population of cells (with 2 distinct mean fluorescence intensity) was observed in all S-MSCs obtained from 7 CLI-patients (data not shown).

Taken together, mRNA and protein levels of CD31, Ve-Cadherin, VEGF R2 and CXCR4 were not significantly increased in S-MSCs. On the contrary, VCAM1 was up-regulated in S-MSCs.

#### Cell secretome

Therapeutic potential of undifferentiated MSCs can be attributed to their capacity to secrete bioactive factors, which may potentially affect both local and systemic physiological processes. Thus, we investigated the effects of EGM-2 medium on the secretome of S-MSCs at day 28 in comparison with MSCs (Table 2). The levels of FGF2, EGF and IGF-1 measured in the S-MSCs culture supernates were significantly lower than in cell-free EGM-2 culture media. A similar pattern was observed in IGF-1 level in the MSCs culture supernates in comparison with cell-free CFU-F culture media. PDGF-AA, Angio-1, LIF-1, CXCL12, IL-6 and HGF are absent from CFU-F and EGM-2 media. Both S-MSCs and MSCs secreted HGF and VEGF-A at a comparable level. In opposite, S-MSCs secreted significantly higher PDGF-AA (22.9-fold higher), Angio-1 (5.6-fold higher), LIF-1 (3.1-fold higher) and lower IL-6 (11.8-fold lower) than MSCs. Interestingly, S-MSCs completely lost their ability to secrete CXCL12.

**Table 2:** Secretome

	CFU-F medium		EGM-2 medium		p^b^
	CFU-F medium^a^	MSCs culture supernates	EGM-2 medium^a^	S-MSCs culture supernates	
VEGF-A (pg/mg proteins)	∅	272 [182–478]	180	464 [288-627]	NS^c^
FGF2 (pg/mg proteins)	∅	∅	101	7 [4–16]	0.003
EGF (pg/mg proteins)	∅	∅	5056	1643 [736–3710]	0.003
IGF-1 (pg/mg proteins)	332	154 [35–308]	826	319 [150–452]	0.003^c^
PDGF-AA (pg/mg proteins)	∅	0.9 [nd–13]	∅	20 [3–60]	0.003
Angio-1 (pg/mg proteins)	∅	159 [91–377]	∅	899 [371–2234]	0.003
LIF (pg/mg proteins)	∅	49 [31–82]	∅	149 [99–283]	0.003
CXCL12 (pg/mg proteins)	∅	3475 [1418–6987]	∅	∅	0.003
IL-6 (pg/mg proteins)	∅	741 [479–1202]	∅	63 [4–127]	0.003
HGF (pg/mg proteins)	∅	17 [1–44]	∅	4 [nd–159]	NS

A set of ten growth factors [VEGF-A, EGF, FGF2, IGF-1, Angio-1, IL-6, HGF, PDGF-AA, LIF, SDF-1 (or CXCL12)] was measured in the MSCs (n = 7) and S-MSCs (n = 7) culture supernates at day 28 and in CFU-F and EGM-2 media

*NS* not significant, ∅ undetectable

^a^Cell-free media

^b^Comparison of MSCs culture supernates versus S-MSCs culture supernates

^c^Comparison after subtraction of basal medium content

**Fig. 4:**
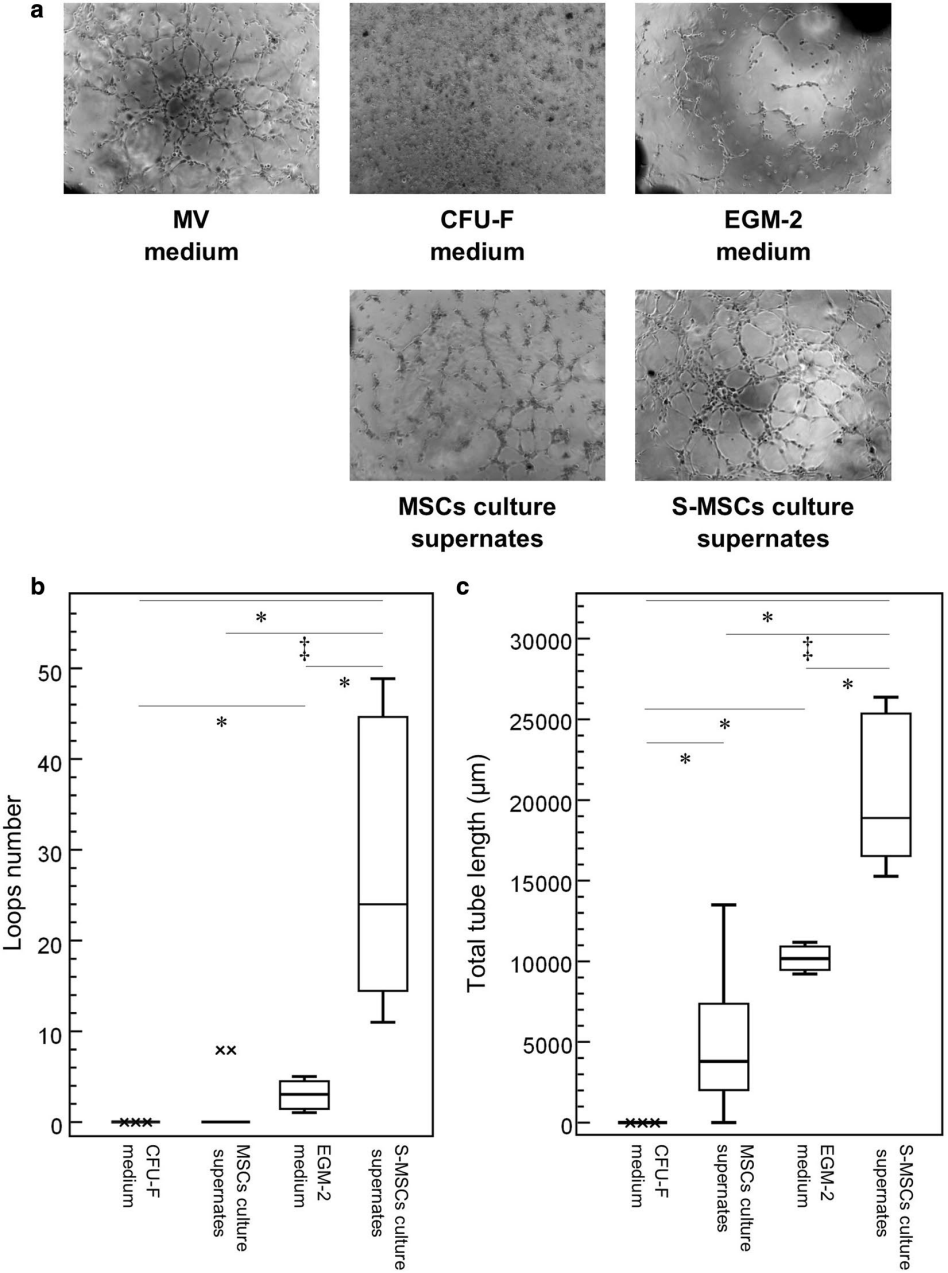
Angiogenic effect of culture supernates. **a** MSCs and S-MSCs culture supernates and cell-free media (CFU-F and EGM-2) were recovered at day 28. HMEC-1 were suspended in endothelial cell growth medium MV (n = 3), in cell-free CFU-F medium (n = 3), in cell-free EGM-2 medium (n = 3), in MSCs culture supernates (obtained from 5 CLI-MSCs, n = 2 each group), in S-MSCs culture supernates (obtained from 5 CLI-S-MSCs, n = 2 each group) and incubated on Matrigel during 24 h. The extend of the network of the capillary-like tubes was appreciated at 3:30 h. **b** Quantification of the loops number at 3:30 h. **c** Quantification of total tube length (µm) at 3:30 h. x: aberrant distribution values as indicated by Box and Whiskers plots Medcalc version 7.3 software (*p < 0.05, ^†^p < 0.01 and ^‡^p < 0.001)

HMEC-1 suspended in endothelial cell growth medium MV were able to form pseudo-tubes in a Matrigel assay (Positive control) (Fig. 4a. HMEC-1 did not form any pseudo-tubes when suspended in CFU-F medium. When suspended in MSCs culture supernates, HMEC-1 could form short tubes lacking branching points and loops. On the contrary, HMEC-1 could generate a network of pseudo-tubes when suspended in S-MSCs culture supernates. This network was significantly more developed when HMEC-1 were suspended in S-MSCs in comparison with MSCs culture supernates (p < 0.0002) but also with cell free EGM-2 medium (p < 0.013).

Taken together, our results indicated that MSCs and S-MSCs can be distinguished by their secretome profiles in terms of growth factors productions and also in terms of functional capacity to form tube-like structures.

#### Cell functional assay: in vitro tube formation assay

Because MSCs and S-MSCs have a proangiogenic potential, we looked for their ability to form capillary-like tube in an in vitro endothelial tube formation assay. Contrary to MRC5 fibroblasts cells line used as negative control, cb-ECFC were constitutively able to form pseudo-tubes in a Matrigel assay. MSCs have low ability for pseudo-tube formation compared with S-MSCs (p value = 0.0008). There was no difference between cb-ECFC and S-MSCs (Additional file 1: Figure S1).

### MSCs and S-MSCs are effective to restore hindlimb blood flow

**Fig. 5:**
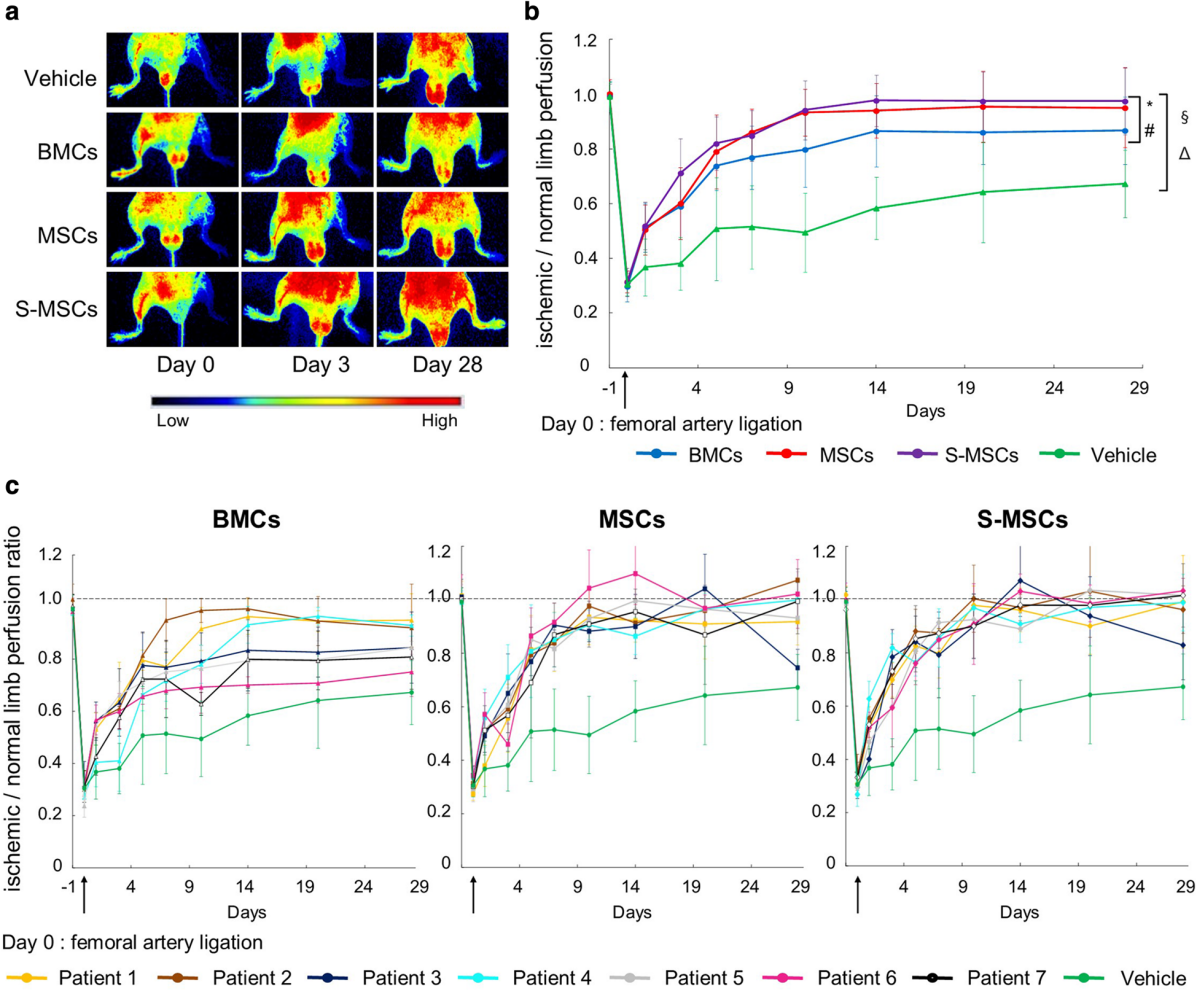
Hindlimb blood flow recovery. **a** Representative LASCA images show improved perfusion in vehicle-, BMCs-, MSCs- and S-MSCs- treated mice at days 0 after ligation, 3 and 28. Low or no blood perfusion was displayed as dark blue, whereas the highest perfusion was displayed as red. **b** Hindlimb blood flow recovery after femoral artery ligation and BMCs (n = 40), MSCs (n = 35), S-MSCs (n = 40), or vehicle (n = 20) injection. CT by BMCs transplantation (blue) was effective in comparison with vehicle (green) injection for blood flow recovery at day 28 but BMCs did not restore completely the blood flow. In contrast MSCs (red) provided complete recovery at day 20 whereas S-MSCs (violet) were effective to completely restore blood flow at day 14. **c** Hindlimb blood flow recovery after BMCs (approximately 6 mice per patient), MSCs (approximately 6 mice per patient) and S-MSCs (approximately 6 mice per patient) infusion. BMCs were obtained from 7 different CLI-patients (Table 1) and were considered as “gold standard” (*comparison of MSCs group versus BMCs group; ^#^S-MSCs versus BMCs; ^¥^S-MSCs versus MSCs; ^§^BMCs versus vehicle; ^Δ^MSCs versus vehicle; ^∞^S-MSCs versus vehicle)

As MSCs and S-MSCs may have a stronger angiogenic potential than BMCs, we compared these cells with vehicle in ischemic *Nude* mice (Fig. 5). The mouse model was validated by the fact that the targeted ischemic ratio was obtained and was identical in each group at baseline (0.99 ± 0.05) and after surgery (0.31 ± 0.04). Figure 5b shows statistical differences between groups. Whatever the type of cells, their injection was more effective to restore leg perfusion as measured by PeriCam^®^ than vehicle (p < 0.0001). MSCs and S-MSCs transplantation (Fig. 5b) allowed significant improvement of flow in comparison with BMCs [p value (MSCs vs. BMCs) < 0.0033; p value (S-MSCs vs. BMCs) < 0.0001]. However, complete flow recovery after BMCs transplantation was not achieved at day 28. In comparison, MSCs allowed to fully restore the flow at day 20. S-MSCs were more effective than BMCs and MSCs and allow achieving full recovery at day 14. Figure 5c shows the heterogeneity in terms of hindlimb blood flow recovery when BMCs are transplanted. In contrast, the response to MSCs and S-MSCs was homogeneous regardless the CLI-patient origin.

These results indicate that MSCs and S-MSCs are more effective than BMCs (and vehicle) to restore hindlimb blood flow.

### S-MSCs improves limb survival in comparison with MSCs

**Fig. 6:**
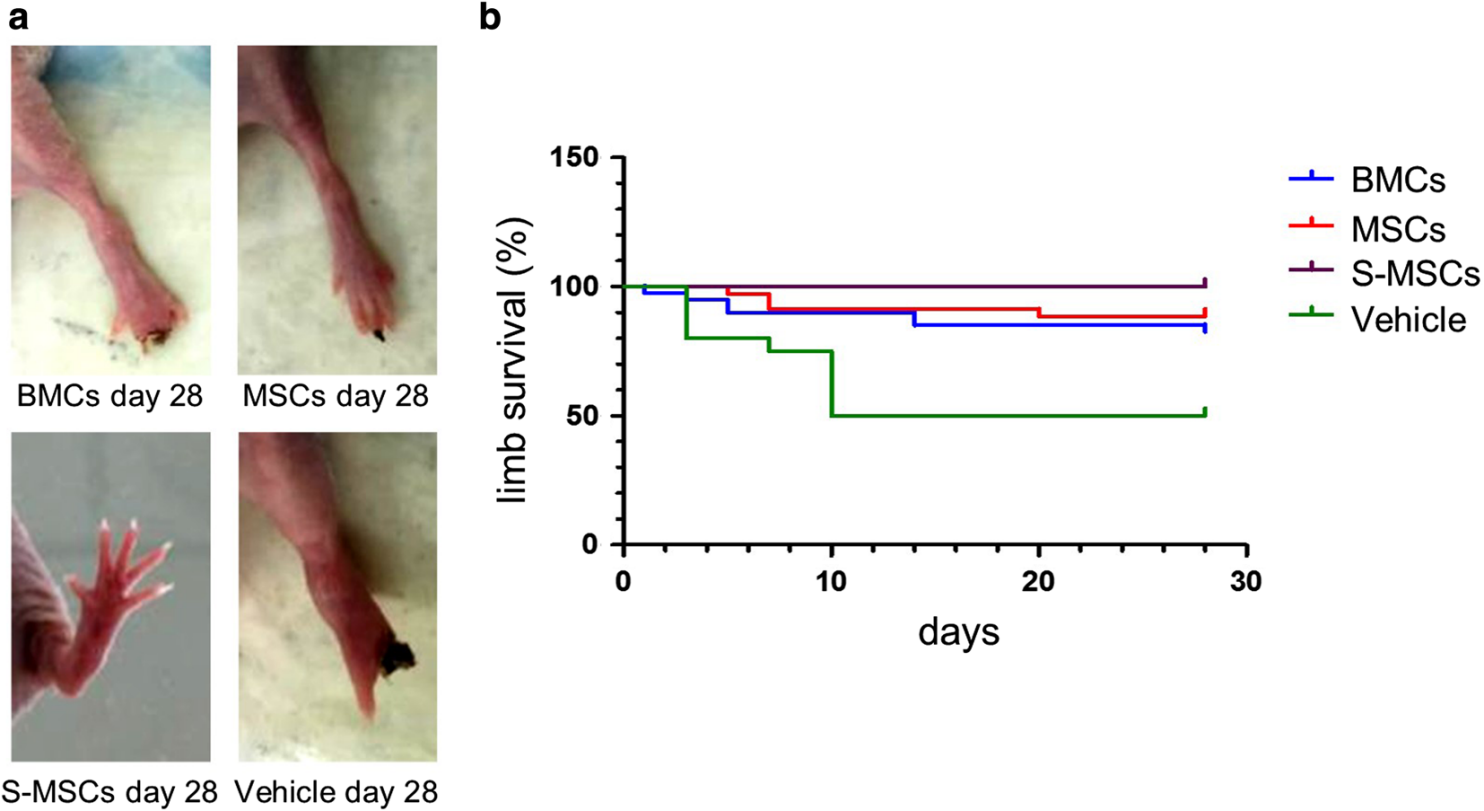
Limb survival. **a** S-MSCs provide complete limb salvage in comparison with MSCs and BMCs. **b** In comparison with vehicle (n = 20), BMCs (n = 40) provided better limb salvage (p < 0.001). There was no difference between BMCs and MSCs (n = 35) treatment. S-MSCs (n = 40) were the most protective compared to BMCs and MSCs (p < 0.05)

Animal limbs were regularly observed during 28 days post operatively to detect necrosis. Ten out of 20 untreated mice developed necrosis as early as day 3 after ligation (Fig. 6). In contrast, in the BMCs group, necrosis affected 7 out of 40 (17.5%) mice. The efficacy of BMCs injection on limb survival was significant compared with vehicle (p < 0.001). In the MSCs group, necrosis was observed in 4 out of 35 (11.4%) mice. MSCs significantly improved limb salvage in comparison with vehicle (p < 0.001) but did not significantly differ from BMCs. In contrast, no necrosis was observed after S-MSCs injection, the necrosis rate being significantly reduced in comparison with BMCs but also with MSCs (p < 0.05).

Taken together, these results suggest that S-MSCs have the strongest angiogenic potential in comparison with BMCs but also MSCs.

### MSCs and S-MSCs injection improves clinical recovery

**Fig. 7:**
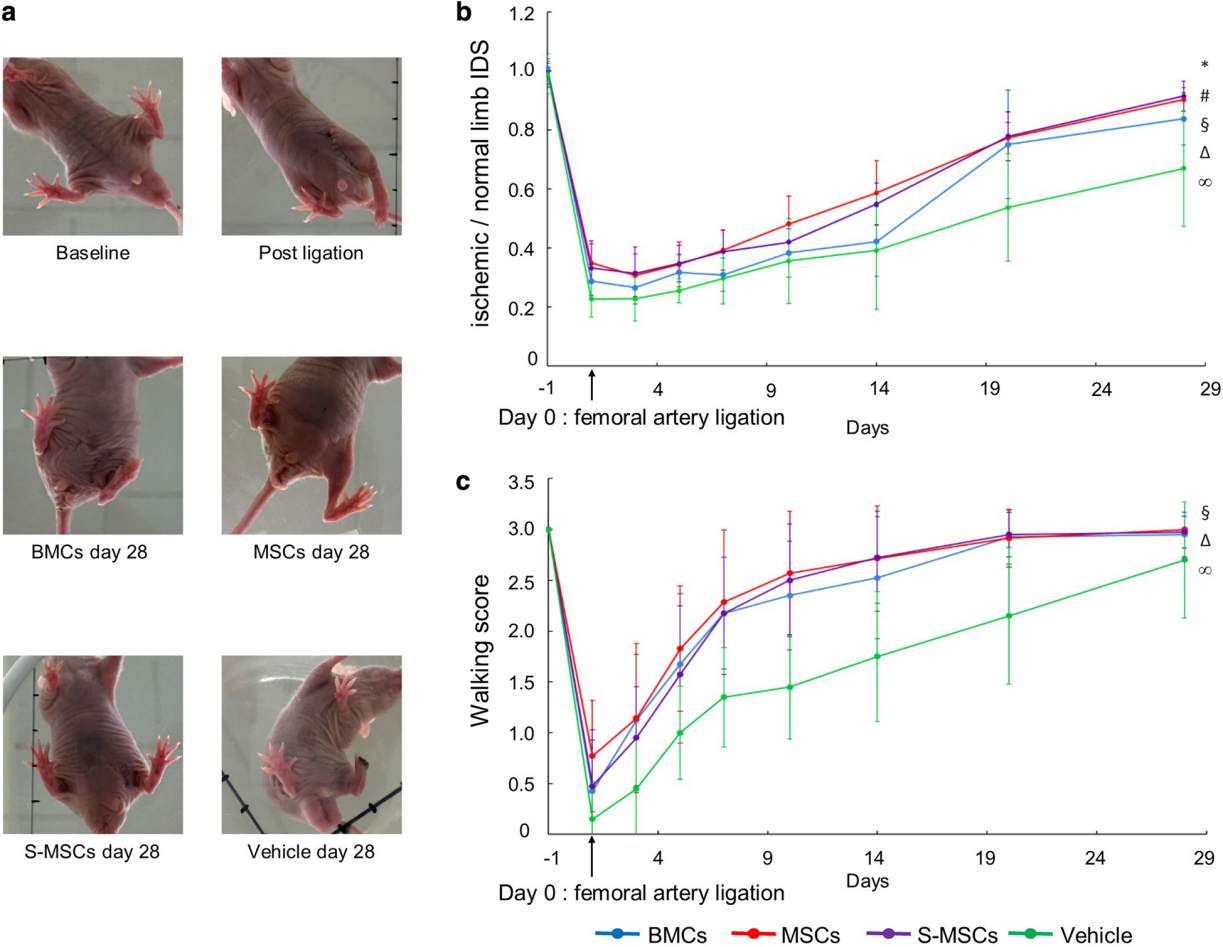
Functional tests (static and dynamic). **a**, **b** Static tests: MSCs and S-MSCs were significantly more effective than BMCs and vehicle to improve IDS. MSCs (red) and S-MSCs (violet) provided significantly higher ratios at day 28 in comparison with BMCs (blue). There was no difference between the MSCs and S-MSCs groups. **c** Dynamic test: S-MSCs, MSCs and BMCs were effective to restore a normal walk in comparison with vehicle [*comparison of MSCs group (n = 35) versus BMCs group (n = 40); ^#^S-MSCs (n = 40) versus BMCs; ^¥^S-MSCs versus MSCs; ^§^BMCs versus vehicle (n = 20); ^Δ^MSCs versus vehicle; ^∞^S-MSCs versus vehicle]

Cell injection using the three types of cells significantly improved clinical recovery in comparison with control group (Fig. 7). In all groups, static and dynamic tests were well-correlated (Pearson’s test: r = 0.6, p < 0.0001).

In order to appreciate recovery, we measured IDS of the ischemic and of the controlateral non-ischemic paw, allowing us to calculate an IDS ratio (equal to 1 to achieve full recovery) (Fig. 7a). BMCs significantly improved IDS ratio evaluated at day 28 in comparison with vehicle (IDS ratio BMCs: 0.84 ± 0.15; vehicle: 0.67 ± 0.20, p < 0.001). MSCs and S-MSCs were significantly more effective than BMCs on IDS ratios at day 28 [IDS ratio MSCs: 0.90 ± 0.11; S-MSCs: 0.93 ± 0.09; p (MSCs vs. BMCs) < 0.05; p (S-MSCs vs. BMCs) < 0.001; MSCs vs. S-MSCs = non-significant) (Fig. 7b).

Cell injection of BMCs, MSCs or S-MSCs significantly improved the walking of the ischemic mice as assessed by the dynamic test (p < 0.0001) (Fig. 7c). Indeed, in the absence of cell infusion, the walking score of the ischemic mice progressively improved barely achieving full recovery at day 28. On the contrary, full recovery was obtained at day 20 after cell infusion, whatever the type of cells used.

### S-MSCs improve angiogenesis and arteriogenesis in ischemic muscle

**Fig. 8:**
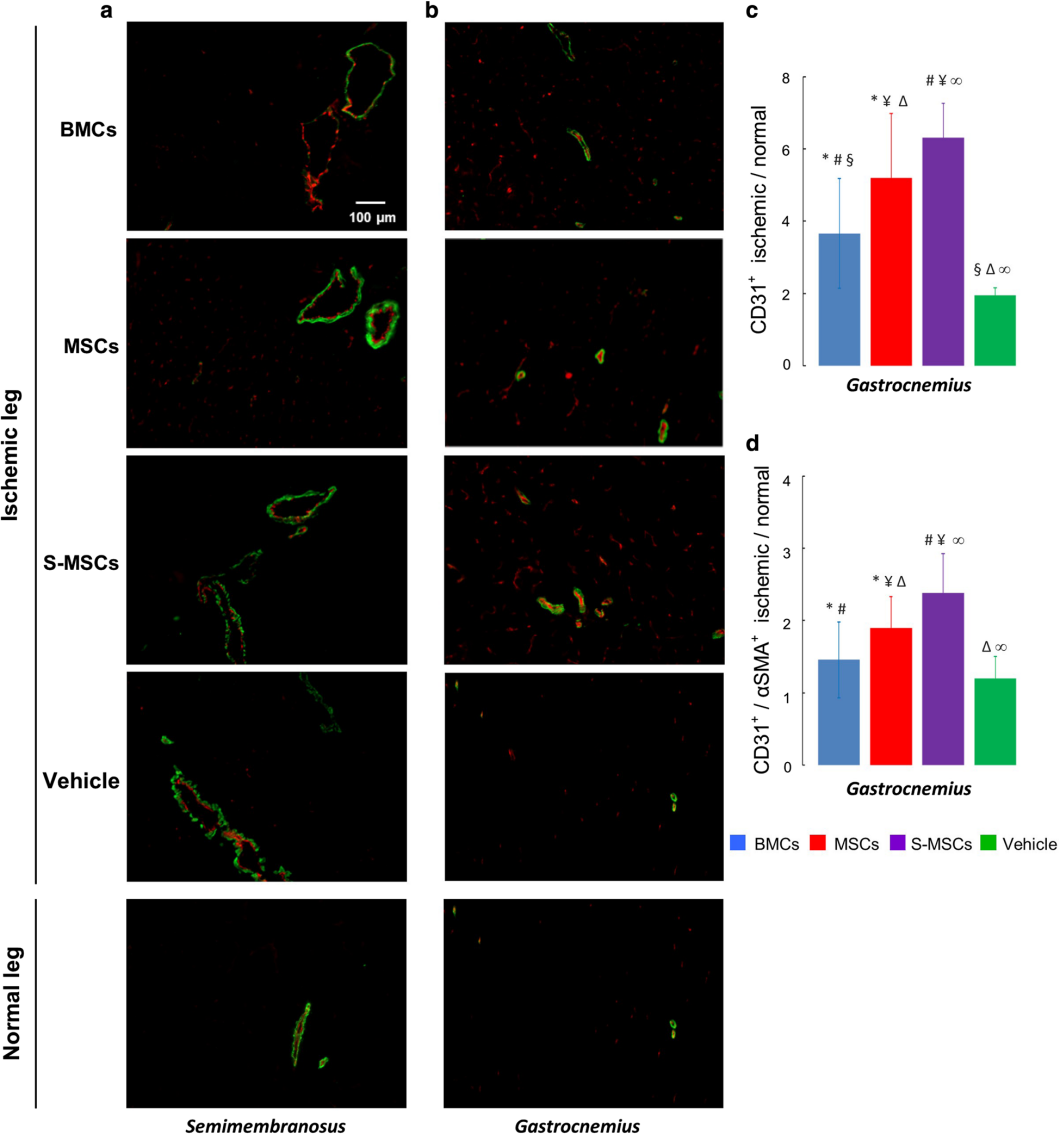
Neovessels visualization with CD31 and αSMA labelling. CD31 + staining (red) detects both angiogenic capillaries and arterioles. αSMA+ (green) detects arterioles. **a** Visualization of capillaries and arterioles in the *semimembranosus* in normal and ischemic legs. **b** Visualization of capillaries and arterioles in the *gastrocnemius* in normal and ischemic legs. **c** Angiogenesis analysis: fluorescence intensity ratio (CD31+ labelling) in the ischemic *gastrocnemius* in comparison with controlateral muscle. **d** Arteriogenesis analysis: Fluorescence intensity ratio (αSMA+/CD31+ labelling) in the ischemic *gastrocnemius* in comparison with controlateral muscle (*comparison of MSCs group versus BMCs group; ^#^S-MSCs versus BMCs; ^¥^S-MSCs versus MSCs; ^§^BMCs versus vehicle; ^Δ^MSCs versus vehicle; ^∞^S-MSCs versus vehicle)

In order to visualize neoangiogenesis after cell infusion, capillary density in the *semimembranosus* (Fig. 8a) and *gastrocnemius* (Fig. 8b) muscles was assessed by immunofluorescence after CD31 labelling. A ratio was calculated from the fluorescence intensity (CD31+) in the ischemic *gastrocnemius* muscle in comparison with the non-ischemic controlateral muscle (Fig. 8c). Capillary density was the highest after S-MSCs injection (S-MSCs fluorescence ratio: 6.31 ± 0.96; p < 0.05 vs. MSCs) (Fig. 8c).

In order to visualize arteriogenesis, we looked at the presence of smooth muscle vascular cells associated with vessels in the *gastrocnemius* and *semimembranosus* muscles (Fig. 8a, b). For this, doubly labelled arterioles (CD31+/αSMA+) were assessed. The analysis of the ischemic *semimembranosus* muscle revealed that the CD31 and αSMA-positive vessels had a larger lumen diameter than the arterioles observed in controlateral normal muscle (Fig. 8a) and that in *gastrocnemius* muscles (Fig. 8b). In *gastrocnemius* muscles, the infusion of either BMCs, MSCs or S-MSCs did not change the aspect of the vessels in comparison with vehicle (Fig. 8b). A ratio was calculated from the fluorescence intensity (CD31+/αSMA+) in the ischemic *gastrocnemius* muscle in comparison with the non-ischemic controlateral muscle (Fig. 8d). The injection of BMCs did not increase the number of CD31 and αSMA-positive vessels in comparison with vehicle (Fig. 8d). In contrast, MSCs treated mice developed a higher rate of CD31 and αSMA-positive cells in comparison with BMCs (1.92 ± 0.36 vs. 1.46 ± 0.53, p < 0.05). Interestingly, S-MSCs treated mice presented with the highest CD31 and αSMA-positive pattern (S-MSCs fluorescence ratio: 2.38 ± 0.54; p < 0.05 vs. MSCs). These results suggest that MSCs and S-MSCs can induce CD31 and αSMA-positive vessels whereas BMCs cannot.

As inflammation is a strong modulator of angiogenesis, we evaluated the macrophage infiltration in response to cell infusion using a CD68 labeling. We found no difference in CD68-positive cell counts after the injection of either type of cells BMCs, MSCs and S-MSCs. Still, there was a difference between either cell type in comparison with vehicle (fluorescence ratio BMCs: 8.72 ± 3.06; MSCs: 9.26 ± 3.37; S-MSCs: 9.71 ± 3.54; vehicle: 12.1 ± 2.18, p value for each type of cells vs. vehicle: p < 0.05).

### S-MSCs favor muscle repair after ischemia

**Fig. 9:**
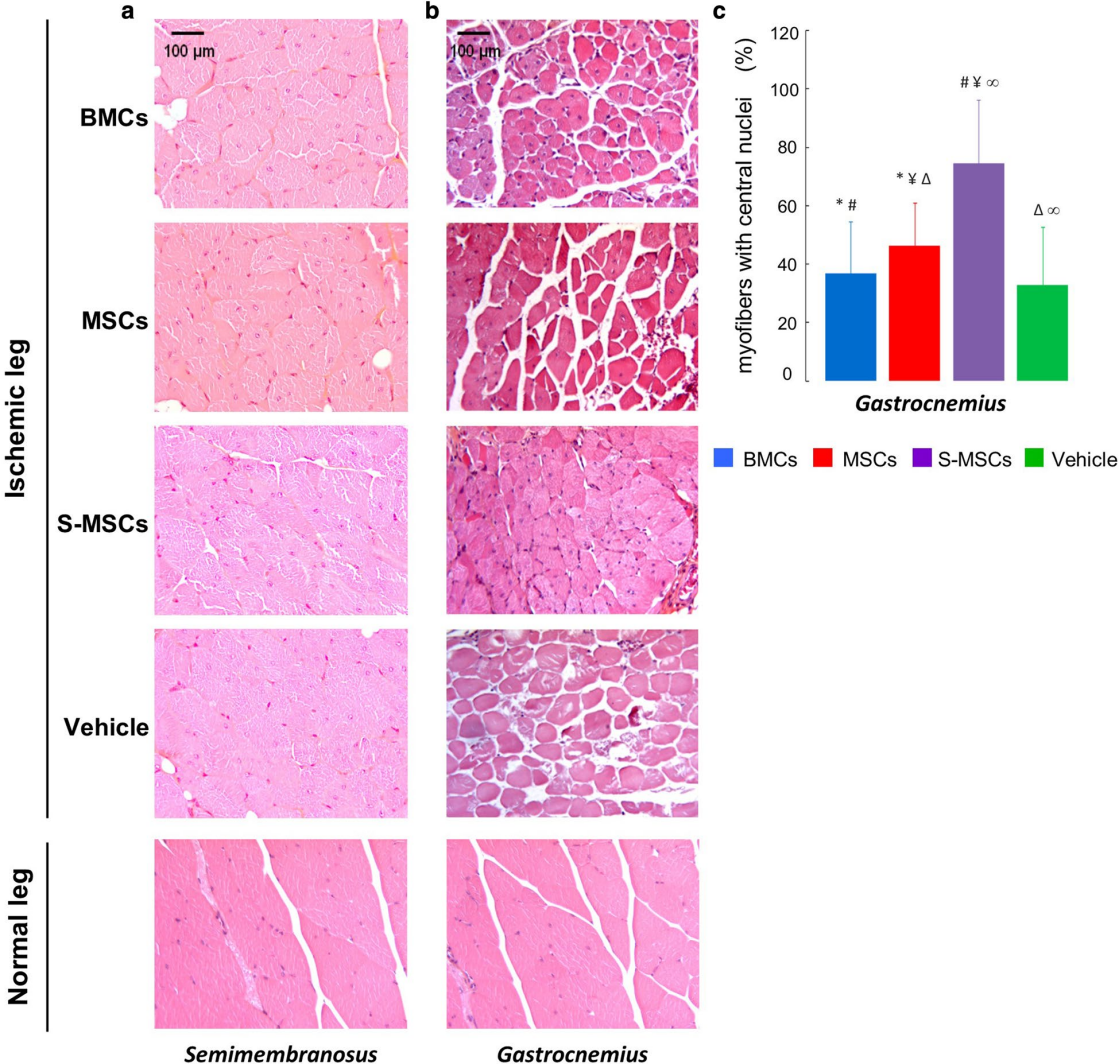
Evaluation of muscle repair. **a** Histological analysis of *semimembranosus* muscle in normal and ischemic leg. **b** Histological analysis of *gastrocnemius* muscle in normal and ischemic leg. **c** Evaluation of muscle repair: percentage of muscle fibers with a central nucleus quantified in the ischemic *gastrocnemius* in comparison with its controlateral muscle (*comparison of MSCs group versus BMCs group; ^#^S-MSCs versus BMCs; ^¥^S-MSCs versus MSCs; ^§^BMCs versus vehicle; ^Δ^MSCs versus vehicle; ^∞^S-MSCs versus vehicle)

A histological analysis of *semimembranosus* (Fig. 9a) and *gastrocnemius* (Fig. 9b) muscles was performed to visualize ischemic muscle damage and muscle repair after cell infusion. For this, the percentage of muscle fibers with a central nucleus was determined. Ligation provoked ischemic lesions which were still visible at day 28 in both muscles. In the ischemic *semimembranosus*, the muscle damage was comparable whatever the type of infused cells (Fig. 9a). In contrast, in the ischemic *gastrocnemius,* there was a glaring difference depending on the type of infused cells (Fig. 9b). BMCs failed to favor *gastrocnemius* repair (37 ± 18% expressed as a percentage of total muscle fibers), in comparison with vehicle (33 ± 20%) (Fig. 9c). MSCs did increase the rate of myofibers with central nuclei (46 ± 15%), in comparison with vehicle (p < 0.01).The effect of S-MSCs on muscle repair (74 ± 20%) was significantly stronger than vehicle (p < 0.001) and BMCs (p < 0.001). Interestingly, S-MSCs were more effective than MSCs on ischemic muscle repair (p < 0.01). This indicates that S-MSCs may favor *gastrocnemius* repair more efficiently than MSCs after ischemia.

### Absence on human nucleus at the end of the experiment

At day 28, a real time qPCR was performed on DNA extracted from *gastrocnemius* muscles in order to detect human nucleus. Human DNA was used as positive control (Ct of human Factor V gene = 24.1 ± 0.1). *Gastrocnemius* muscles of vehicle group and non-ischemic controlateral muscles did not contain any detectable human DNA (Ct of human Factor V gene > 35). No human DNA (Ct of human Factor V gene > 35) was found in ischemic and injected *gastrocnemius* muscles, whatever the type of cells (BMCs, MSCs or S-MSCs). This suggests the absence of any residual human cell at the end of the experiment.

## Discussion

Among innovative therapies, CT is a good candidate to treat patients with severe PAD. BMCs are considered as “gold standard” in CLI-CT because it was used by the founder trial [4]. In spite of encouraging clinical studies, the efficacy of CT remains controversial [7, 8]. This may be partially explained by the heterogeneity of autologous BMCs [11]. In such BMCs, HSCs are extremely rare and may not support proangiogenic properties of BMCs [5]. Still, these autologous BMCs may be the source of other types of proangiogenic stem cells. Indeed undifferentiated MSCs were obtained from BMCs CLI-patients as previously described [11, 29, 36].

MSCs are well-recognized for their high proliferation and differentiation potential. MSCs support a significant paracrine effect through the secretion of proangiogenic cytokines which provide anti-apoptotic effects and stimulate revascularization [15].

In the first step of our study, we confirmed the presence of MSCs in CLI-BMCs. We next evaluated if the culture of MSCs in a medium enriched in endothelial growth factors (EGM-2) could improve the proangiogenic properties of MSCs that we named S-MSCs. S-MSCs that we obtained from CLI-patients showed a morphology which was indistinguishable from MSCs obtained in CFU-F medium. However, S-MSCs’ doubling time was shorter for when compared with MSCs’, in agreement with published data [21, 25, 37].

We next characterized S-MSCs in FC and observed that these cells did not express endothelial markers (CD31, Ve-Cadherin, VEGF R1 and VEGF R2). Using other sources of MSCs derived from umbilical cord blood [25, 26], adipose tissue [22, 23, 27] or BM, but from healthy donors [21], an endothelial differentiation of MSCs was reported when cultured in EGM-2 medium [37]. Oswald et al. was the first to report that a VEGF induction gave rise to the endothelial differentiation of MSCs expression classical endothelial markers but not CD31 [38]. Tancharoen et al. also, showed that S-MSCs expressed endothelial markers including CD31 but with a much lower level than HUVECs [39]. In agreement with our data, other published studies on WJ-MSCs [32], BM-MSCs [40] or diabetic patients ADSCs [41], stated that endothelial cell growth supplement alone could not per se induce the expression of molecular markers of ECs. This illustrates the difficulty encountered to define such cells when cultured in different conditions. The term of “endothelial-like cells” has been employed by some authors [21, 32, 40, 42] in spite of a high heterogeneity in both culture conditions and MSCs origin. Interestingly, it was suggested that shear stress could be a major parameter for endothelial differentiation [40].

Our data show that MSCs and S-MSCs express comparable proteomic and transcriptomic profiles except for VCAM1 which is significantly higher in S-MSCs. VCAM1 mediated the interaction of MSCs with ECs, which is essential for MSCs homing [43]. This expression of VCAM1 was previously reported in S-MSCs obtained in the presence of VEGF-A [38]. A role of EGF, which is present in EGM-2 medium, has been shown to increase VCAM1 and MSCs adhesion to ECs [44].

To further characterize S-MSCs, we analyzed their secretion capacity. For this, we quantified a set of ten growth factors in S-MSCs culture supernates in comparison with MSCs. Our data indicate major differences between these two types of cells: in comparison with MSCs, S-MSCs produce high levels of PDGF-AA, Angio-1 and LIF whereas the secretion of pro-inflammatory IL-6 is significantly lower. CXCL12 is not secreted by S-MSCs whereas it is found at high concentration in the MSCs supernatant. The loss of capacity of S-MSCs to secrete CXCL12 could be the consequence of EGM-2 induction. Using a supplementation with platelet-rich plasma, which contains PDGF, TGF*β*1, FGF2, IGF-1, VEGF and EGF (some of them being also present in EGM-2 medium), Goedecke et al, have reported a loss of CXCL12 secretion associated with a defect in HSCs migration [45]. The analysis of S-MSCs supernatants indicated the diminution in the concentration of FGF2, EGF and IGF-1 present in the EGM-2 medium. This may be the consequence of a consumption or could be explained by the endocytosis of a ligand-receptor complex.

In order to further characterize the secretome, we evaluated the capacity of supernates to induce tube-like structures from HMEC-1. We observed that S-MSCs culture supernates had the strongest ability to induce tube-like structures. This is in agreement with the results of growth factor quantification showing that S-MSCs supernates contain higher concentrations of proangiogenic factors. Taken together, these results suggest that the secretome analysis allows to differentiate S-MSCs from MSCs. A further issue would be to elucidate the potency of each growth factor present in conditioning media. Furthermore, the existence of possible autocrine mechanisms has to be considered.

Our study shows that S-MSCs can be functionally distinguished from MSCs by their stronger capacity to form pseudo-tubes in vitro. This confirms most reports having used EGM-2 induction [21–23, 25, 40, 41, 46]. However, Choi et al, raised the point that MSCs are able to form tube-like structures in Matrigel but the observation of these pseudo-tubes by electron microscopy revealed the absence of lumen [32]. Although commonly used, Matrigel cannot be considered as a specific angiogenic assay [47].

It was therefore mandatory to evaluate in vivo, the proangiogenic potential of MSCs and S-MSCs. In a well-established mouse model of HLIM [30], our results clearly indicate that MSCs from CLI-patients completely restore blood flow (primary endpoint) in comparison with BMCs that we considered as the “gold standard”. Such results are in agreement with those obtained by Iwase et al, who concluded that BM-MSCs were superior to BMCs in promoting neovascularization [48]. The limit of this study was that BM-MSCs were obtained from young rats. Indeed, it has been shown that age could impair stem cell properties [9]. In the context of CLI, this alteration could be further increased in case of associated risk factors [20]. In contrast, MSCs obtained from CLI-patients conserve proangiogenic properties through a paracrine mechanism [15, 29, 49]. This may be at least partly explained by the fact that, BM-MSCs from CLI-patient have a similar secretome profile compared to that of healthy MSCs [29, 50]. In agreement with this hypothesis, Smadja et al, 2012 and Gremmels et al, 2014 showed, in a HLIM model, that MSCs from CLI-patients had a comparable proangiogenic potential to MSCs isolated from age matched patients free of any cardiovascular disease [29, 36].

To our knowledge, this is the first report to establish the property of S-MSCs obtained from CLI-patients infused in HLIM model. Interestingly, S-MSCs restored completely blood flow and earlier than MSCs. The infusion of BMCs led to a highly heterogeneous response in terms of extend and kinetics of blood flow recovery. This fits with the heterogeneity of BMCs in terms of cellular content. In contrast, the infusion of MSCs and S-MSCs restored blood flow in a homogeneous manner. The selection and amplification in culture may allow to obtain a consistent favorable response to infusion, and this whatever the patient’s clinical status.

We further demonstrated that MSCs and S-MSCs could improve clinical recovery as well as limb salvage. In agreement with Iwase et al, MSCs were more effective than BMCs to save limb [48]. Our results indicate that S-MSCs were also more effective than MSCs.

Interestingly, the analysis of the *gastrocnemius* muscle clearly shows that S-MSCs not only enhance neovascularization but also favor the development of vascular muscle cells suggesting the formation of mature and stable capillaries [51]. Indeed, arteriogenesis is more capable of restoring tissue blood supply than angiogenesis [52]. Collateral vessels have the capacity to carry a larger volume of blood than sprouting capillary networks [53].

In contrast, the analysis of the *semimembranosus* muscle indicates an enlargement of the vessels and limited ischemia. This is explained by the anatomic situation of this muscle which is located proximally to the ligation site. It was reported that, following a femoral artery ligation, an arteriogenic process is initiated by the increase of blood pressure in the pre-existing collaterals that circumvent the obstruction and can supply blood flow to the distal tissue [54]. In contrast, *gastrocnemius* muscle being downstream from the ligation site is more sensitive to ischemia and therefore prone to undergo a process of angiogenesis. In this respect, our results clearly show the favorable effect of cell infusion. This effect may be more the consequence of a paracrine effect considering the high potential of secretion of such cells. The proangiogenic effect is unlikely related to endothelial differentiation of infused cells since no human DNA was detected at the end of the experiment. Previous studies have illustrated the rapid disappearance of infused cells [55, 56].

A major advantage of S-MSCs may be their capacity to improve skeletal muscle repair after ischemia. This effect could explain the efficacy of S-MSCs to reduce the amputation rate. To evaluate muscle repair, we quantified myofibers with central nucleus location. Indeed, muscle regeneration is characterized by the activation of myogenic cells which leads to the formation of new myofibers. These fibers are recognized by a centrally located myonucleus [34, 35, 57, 58].

MSCs have been evaluated in 13 clinical trials in the last decade [17–19, 59–66] and have included a total population of 216 MSCs-treated patients. These studies have established the safety of such cells [18, 67], which may be at least partly explained by their immunomodulatory properties [16, 68, 69]. Clinical trial results were in favor of efficacy, one randomized clinical trial concluding that MSCs were more effective than BMCs [17].

Our study pointed out the importance of the secretome. This should be extended to the analysis of secretome content including extracellular vesicles, exosomes, microRNAs, mRNAs, long non-coding RNAs, circular RNAs which may have an interest in CLI-therapeutics [15, 70].

## Conclusions

MSCs have been suggested to be a promising alternative source for ischemic diseases [42].

The present study shows that S-MSCs can be obtained from the BM of patients presenting severe PAD. S-MSCs can restore blood flow as efficiently as MSCs and more potently than BMCs. A major issue of CT in PAD is to provide a fully stable and mature vascular network. In this regard, S-MSCs are promising candidates as they restore flow in CLI and provide muscle repair. Many suggested improvements can be proposed to produce these cells for CLI-cell therapy. MSC secretome may also be of therapeutic value.

## Additional file

**Additional file 1**: **Figure S1.** Cell functional assay: in vitro tube formation assay. **a** MRC5 (n = 5), cb-ECFC (n = 4), MSCs (n = 7) and S-MSCs (n = 7) were incubated on Matrigel. The extend of the network of the capillary-like tubes was appreciated at the time of maximal network. **b** S-MSCs form more cord-like structures than MSCs (cb-ECFC are used as positive control and MRC5 as negative control). The extend of the network of the capillary-like tubes was appreciated at the time of maximal network by the quantification of the loops number. (^†^p < 0.01 and ^‡^p < 0.001).

## Data Availability

The data supporting the conclusions of this article are included within the article.

## Abbreviations

ADSCs: adipose tissue-derived stem cells
Angio-1: angiopoietin-1
BALI: bone marrow autograft in limb ischemia
BM: bone marrow
BMCs: bone marrow derived cells
cb-ECFCs: cord blood endothelial colony-forming cells
CDH5: Ve-Cadherin (CD144)
CFU-F: colony forming unit-fibroblast
CLI: critical limb ischemia
CT: cell therapy
CXCL12: chemokine CXC motif ligand 12 (or stromal-cell-derived factor-1; SDF-1)
CXCR4: CXC motif receptor 4 (CD184)
ECs: endothelial cells
EGM-2: endothelial basal medium-2
EGF: epidermal growth factor
FC: flow cytometry
FITC: fluorescein
FGF2: fibroblast growth factor-2 (basic FGF)
HE: hematoxylin–eosin
HGF: hepatocyte growth factor
HLA-DR: human leukocyte antigen-D related
HLIM: hind limb ischemia model
HMEC-1: human microvascular endothelium cells
HSCs: hematopoietic stem cells
IDS: interdigital spacing
IGF-1: insulin-like growth factor-1
IL-6: interleukin-6
S-MSCs: stimulated-mesenchymal stem cells
LASCA: laser speckle contrast analysis
LIF: leukemia-inhibitory factor
MCAM: melanoma adhesion molecule (CD146)
MNCs: mononuclear cells
MSCs: mesenchymal stem cells
NO-CLI: “no-option CLI”
PAD: peripheral arterial disease
PBS: phosphate buffer saline
PCR: polymerase chain reaction
PDGF-AA: platelet-derived growth factor, alpha polypeptide
PDGF RA: platelet-derived growth factor receptor alpha (CD140a)
PDGF RB: platelet-derived growth factor receptor beta (CD140b)
PE: phycoerythrin
PECAM1: platelet endothelial cell adhesion molecule-1 (CD31)
PLTs: platelets
RT-qPCR: reverse transcriptase-quantitative real-time polymerase chain reaction
αSMA: alpha-smooth muscle actin
TcPO_2_: transcutaneous partial pressure of oxygen
VCAM1: vascular cell adhesion molecule 1 (CD106)
VEGF: vascular endothelial growth factor
VEGF R: vascular endothelial growth factor receptor
vWF: von Willebrand factor
WJ-MSCs: umbilical cord Wharton’s jelly-derived mesenchymal stem cells
HLIM: hind limb ischemia model
