# Supplementary figures and images for: In vivo efficacy of endothelial growth medium stimulated mesenchymal stem cells derived from patients with critical limb ischemia
Source: J Transl Med. 2019 Aug 9;17:261. doi: 10.1186/s12967-019-2003-3 (PMC6688282; doi:10.1186/s12967-019-2003-3)

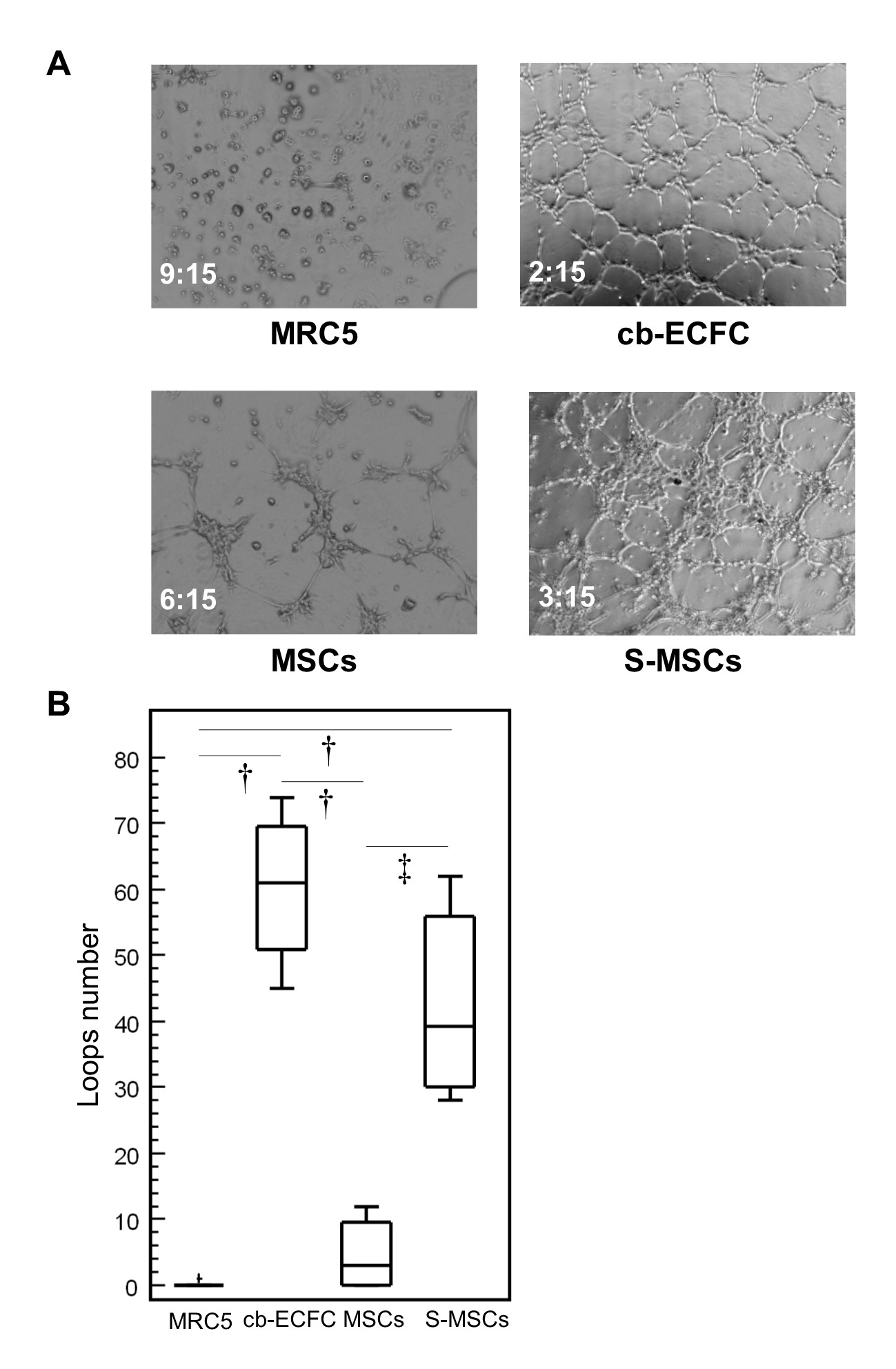

Supplement: Supplementary file 1 — Additional file 1: Figure S1. Cell functional assay: in vitro tube formation assay. a MRC5 (n = 5), cb-ECFC (n = 4), MSCs (n = 7) and S-MSCs (n = 7) were incubated on Matrigel. The extend of the network of the capillary-like tubes was appreciated at the time of maximal network. b S-MSCs form more cord-like structures than MSCs (cb-ECFC are used as positive control and MRC5 as negative control). The extend of the network of the capillary-like tubes was appreciated at the time of maximal network by the quantification of the loops number. (†p < 0.01 and ‡p < 0.001). [file 12967_2019_2003_MOESM1_ESM.jpg]
